# In situ X-ray scattering observation of two-dimensional interfacial colloidal crystallization

**DOI:** 10.1038/s41467-018-03767-y

**Published:** 2018-04-06

**Authors:** Longlong Wu, Xiao Wang, Geng Wang, Gang Chen

**Affiliations:** 1 School of Physical Science and Technology, ShanghaiTech University, Shanghai, 201210 China; 2 Shanghai Institute of Microsystem and Information Technology, Chinese Academy of Sciences, Shanghai, 200050 China; 3 University of Chinese Academy of Sciences, No.19A Yuquan Road, Beijing, 100049 China; 4 Shanghai Synchrotron Radiation Facility, Shanghai Institute of Applied Physics, Chinese Academy of Sciences, Shanghai, 201204 China

## Abstract

Charged colloids at interfaces hold such a simple configuration that their interactions are supposed to be fully elucidated in the framework of classical electrostatics, yet the mysterious existence of attractive forces between these like-charged particles has puzzled the scientific community for decades. Here, we perform the in situ grazing-incidence small-angle X-ray scattering study of the dynamic self-assembling process of two-dimensional interfacial colloids. This approach allows simultaneous monitoring of the in-plane structure and ordering and the out-of-plane immersion depth variation. Upon compression, the system undergoes multiple metastable intermediate states before the stable hexagonal close-packed monolayer forms under van der Waals attraction. Remarkably, the immersion depth of colloidal particles is found to increase as the interparticle distance decreases. Numerical simulations demonstrate the interface around a colloid is deformed by the electrostatic force from its neighboring particles, which induces the long-range capillary attraction.

## Abstract (web summary)

Colloids adsorbed at fluid interfaces can self-assemble into crystal, but the detail remains largely unknown due to experimental challenges. Using in situ X-ray scattering, Wu et al. show that out-of-plane electrostatic force induces in-plane capillary attraction between like-charged particles.

## Introduction

Colloids adsorbed at a fluid interface are ubiquitous in nature and central to a promising route to materials synthesis that combines considerable freedom of material choices with the opportunity to create highly ordered structures on the length scales from nano- to micrometers^1–6^. The two-dimensional (2D) colloidal systems are also of interest in relation to a wide range of phenomena, including self-assembly and phase behavior in colloid science and condensed-matter physics^7–11^. When in equilibrium, colloids will straddle across the interface with their three-phase contact angle described by Young’s law^12^ and develop an asymmetric counterion distribution, which results in a dipole moment normal to the interface. If the interface area is confined, the repulsive Coulomb interactions between colloids can induce ordering and even crystallization^13,14^. However, metastable crystallites and voids have also been observed in the absence of area confinement^15,16^. Neither should be possible in a system with purely repulsive interactions, suggesting that like-charged particles at interfaces can also experience attractive interactions and in turn generate a secondary potential well apart from the primary minimum created by van der Waals attraction^14,16–18^. Since the first direct microscopic observation of 2D colloidal crystals trapped at the air/water interface by Pieranski^19^, considerable experimental and theoretical efforts have been devoted to the study of colloidal particle interaction, dynamics and assembly at liquid interfaces^15,20–23^. Despite some new developments in experiments and the emergence of several theories in recent decades^24–28^, much confusion still surrounds the interfacial crystallization process and the origin of the attractive forces between like-charged colloidal particles^29,30^.

The difficulty partially lies in the limited tools available to investigate the interfacial colloidal system. Most studies are carried out by optical microscopy^31^ and optical tweezer techniques^32^, while the popular methods, such as electron microscopy and atomic-force microscopy, become incompetent at a liquid interface. The optical techniques set the lower limit of colloid sizes suitable for study (in micrometer scale), hence the gravitational effect is immanent^33^. The finite field depth of a microscope objective also leaves it insensitive to the position of particles with respect to the interface. In addition, the substrate-induced confinement effect may present in systems studied on glass slides^14,31^.

We report the in situ grazing-incidence small-angle X-ray scattering (GISAXS) study of the real-time self-assembly process of polystyrene nanospheres (PNSs) on the air/water interface of a Langmuir-Blodgett (LB) trough. This approach allows for simultaneous monitoring of the in-plane ordering and crystallization and the out-of-plane immersion depth (or contact angle) variation, providing a complete picture of the interfacial colloidal self-assembly process. By using X-rays instead of visible light of a microscope, the size of colloids in study can be reduced from micro- to nanometer scale, the dimension has never been explored in situ. Considering the gravitational force is proportional to the third order of a radius, the effect of gravity will be dramatically reduced, rendering it negligible^33^. The substrate confinement effect is further ruled out by conducting measurements on the bulk water surface of an LB trough. Upon compression, multiple metastable intermediate states are observed before the stable 2D hexagonal close-packed superlattice monolayer forms under van der Waals attraction. Surprisingly, the immersion depth of the colloidal particles is found to vary with the interparticle distance. Numerical simulations demonstrate the out-of-plane component of the electrostatic force from neighboring particles increases as the interparticle distance decreases. Such a force presses the particle into water, which deforms the interface and induces the long-ranged capillary attraction. This new finding provides an important clue to the long-standing mystery of the attractive interaction between like-charged particles.

## Results

### Experimental set-up and principle

**Fig. 1:**
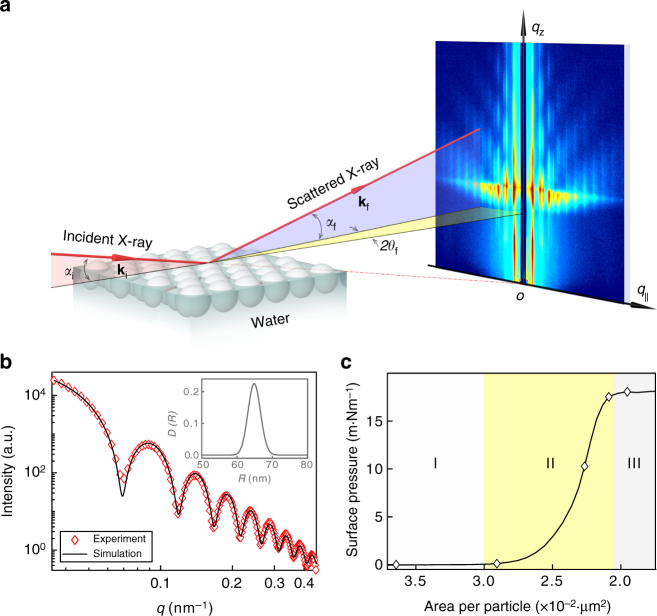
GISAXS experimental set-up and sample characterizations. **a** The schematic illustration of the GISAXS experimental geometry. **k**_i_ and **k**_f_ are the incident and scattered wave vectors, respectively, yielding the wavevector transfer (i.e., the reciprocal space vector) ${\mathbf{q}} = {\mathbf{k}}_{\mathrm{f}} - {\mathbf{k}}_{\mathrm{i}}$; *α*_i_ is the incident angle of X-ray beam; $2\theta _{\mathrm{f}}$ and *α*_f_ are the azimuthal and exit scattering angles, respectively. A long beamstop is installed to protect the detector from the reflected and direct beams. **b** The SAXS data (red diamonds) for PNSs dispersed in aqueous solution and their theoretical fit (black solid line) using the spherical form factor with its polydispersity described by the Schultz distribution. Inset: the size distribution of the PNSs. **c** The real-time surface pressure-particle area isotherm for the self-assembly of PNSs adsorbed at the air/water interface of the LB trough taken during the in situ GISAXS experiment

The experimental set-up and the principle behind GISAXS are presented schematically in Fig. 1a. The synchrotron X-ray beam is directed toward the PNSs floating on the water surface of an LB trough at the grazing angle *α*_i_ = 0.367° (Supplementary Fig. 1). The wavelength of the incident X-ray is 1.239 Å. The charge-coupled device (CCD) detector, placed perpendicular to the incident beam, is used to record the 2D GISAXS patterns. The PNSs in solution are first characterized by small-angle X-ray scattering (SAXS) and the one-dimensional (1D) scattering profile is shown in Fig. 1b. By fitting the SAXS data, the PNSs are found to be 126.4 nm in diameter with the dispersion of ~2.7%, and the similar results are attained from the scanning electron microscopy (SEM) measurements (Supplementary Fig. 2). The surface charge density of the PNSs is *σ*_0_ = 1.34 μC cm^−2^, obtained through the zeta potential measurement. Prior to the GISAXS experiment, the PNS solution is carefully deposited onto the water surface through a tilted silicon wafer. After the water surface stabilizes, the PNSs are compressed through the barriers on the LB trough at a speed of 0.3 mm min^−1^ and the real-time X-ray scattering signal is collected by the CCD detector. In the meanwhile, the surface pressure evolution is monitored by a Wilhelmy plate (Fig. 1c). Overall, the surface pressure (Π)—particle area isotherm can be partitioned into three distinct stages. In stage I, the surface pressure stays nearly constant and only slightly increases due to the long-range electrostatic repulsion between colloidal particles^22^. In stage II, the surface pressure rises steeply, which is caused by the repulsive interactions between particles on compression^34^. In stage III, the surface pressure remains constant at 18.3 mN m^−1^. The locus of rapid slope change situated between stages II and III concurs with the collapse of the PNS superlattice monolayer.

### In situ GISAXS measurements

**Fig. 2:**
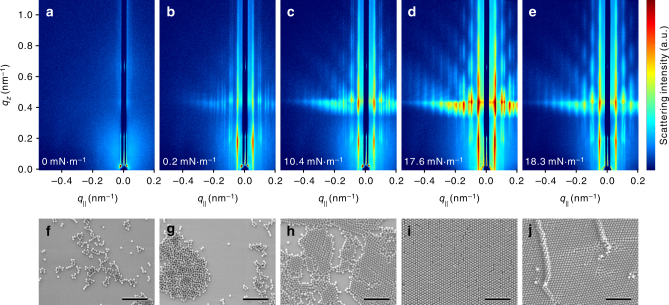
In situ GISAXS and ex situ SEM measurements. **a**–**e** The in situ GISAXS patterns recorded during the self-assembly of PNSs at the air/water interface of the LB trough at the surface pressures of 0, 0.2, 10.4, 17.6, 18.3 mN m^−1^ and the corresponding areas per particle are 3.68 × 10^−2^, 2.82 × 10^−2^, 2.30 × 10^−2^, 2.13 × 10^−2^, and 1.99 × 10^−2^ μm^2^, respectively (marked as diamonds in Fig. 1c). **f**–**j** The ex situ SEM images of the dried PNSs on silicon substrates taken from the LB trough at the same assembling stages as the in situ GISAXS measurements (all the scale bars are 1.5 μm)

Following the PNS self-assembly process, a continuous series of GISAXS patterns are recorded (see Supplementary Movie 1). The five representative scattering patterns (the upper row of Fig. 2) are taken at Π of 0, 0.2, 10.4, 17.6, and 18.3 mN m^−1^, respectively. The corresponding SEM images (the bottom row of Fig. 2) are sampled at the same Π points of the isotherm as the GISAXS measurements. It should be emphasized that the GISAXS data are recorded directly from the air/water interface without any intermediary procedure. Nevertheless, care must be taken in dealing with the SEM data as the sample extracted at a given surface pressure may undergo structural transformation during drying.

At the initial stage, there is only weak scattering signal near the beamstop (Fig. 2a). The PNSs form dimers, trimers…, and strings at this stage as can be identified in Fig. 2f. After short-time compression, the diffraction rods appear in the GISAXS pattern (Fig. 2b) at the scattering wavevector transfer $q_\parallel$ of ±0.051, ±0.089, ±0.102, ±0.135, and ±0.153 nm^−1^. At this stage, large colloidal islands form and coexist with the small clusters from the initial stage (Fig. 2g). The appearance of the distinct scattering peaks reveals the PNSs in the colloidal islands have long-range ordering, however, the radial distribution function (Supplementary Fig. 3) extracted from the corresponding SEM image (Fig. 2g) shows the PNSs are randomly distributed, which underscores the importance of in situ measurements. The difficulty in isolating this intermediate phase ex situ indicates that the long-range interactions between PNSs are mediated through the air/water interface. Under further compression, the PNS clusters and islands collide with each other. The interaction force is anisotropic, which tends to distort the metastable superlattice structure as revealed by the splitting of the main diffraction peaks in Fig. 2c. This state is again not caught in the corresponding SEM image (Fig. 2h). After long-time continuous compression, the area between the LB barriers is fully occupied by the PNSs and the stable 2D hexagonal close-packed monolayer (Fig. 2i) forms whose X-ray diffraction peaks show at $q_\parallel$ of ±0.056, ±0.099, ±0.112, ±0.148, and ±0.168 nm^−1^, respectively (Fig. 2d). Under additional compression, the intensity of the diffraction rods decreases (Fig. 2e), indicating the order of the monolayer declines as shown in Fig. 2j.

### In-plane structure analysis

To quantitatively determine the structure and ordering of the PNSs adsorbed at the air/water interface, the GISAXS data are analyzed in the framework of the distorted-wave Born approximation (DWBA)^35–37^. In this theory, the GISAXS intensity $I_{{\mathrm{GISAXS}}}\left( {q_\parallel ,q_z} \right)$ is proportional to $|T_{\mathrm{i}}|^2|T_{\mathrm{f}}|^2P\left( {q_\parallel ,q_z} \right){\mathrm{S}}\left( {q_\parallel ,q_z} \right)$, where *T*_i_ and *T*_f_ are the Fresnel transmission coefficients of the incident and reflected waves, $P\left( {q_\parallel ,q_z} \right)$ and ${\mathrm{S}}\left( {q_\parallel ,q_z} \right)$ are the form factor and the interference function related to the spatial arrangement of the PNSs, $q_\parallel$ and $q_z$ are the in-plane and out-of-plane components of the wavevector transfer (see Supplementary Note 1). The data analyses are performed separately in terms of $q_\parallel$ and $q_z$ by taking line cuts from the experimental GISAXS patterns and finding their best fits to the theoretical model. For X-ray scattering from a 2D superlattice structure, the Bragg reflection ${{q}}_{{\mathrm{hk}}}$ is generally given by $q_{hk} = 2{\mathrm{\pi }}\sqrt {a^2h^2 + b^2k^2 - 2abhk{\mathrm{cos}}\left( \phi \right)} /ab\sin \left( \phi \right)$, where *a* and *b* are the bases of the unit cell, $\phi$ is the angle between *a* and *b*, and {*hk*} are the Miller indices. Specifically, the 2D hexagonal lattice has *a* = *b* and $\phi = 120^\circ$.

**Fig. 3:**
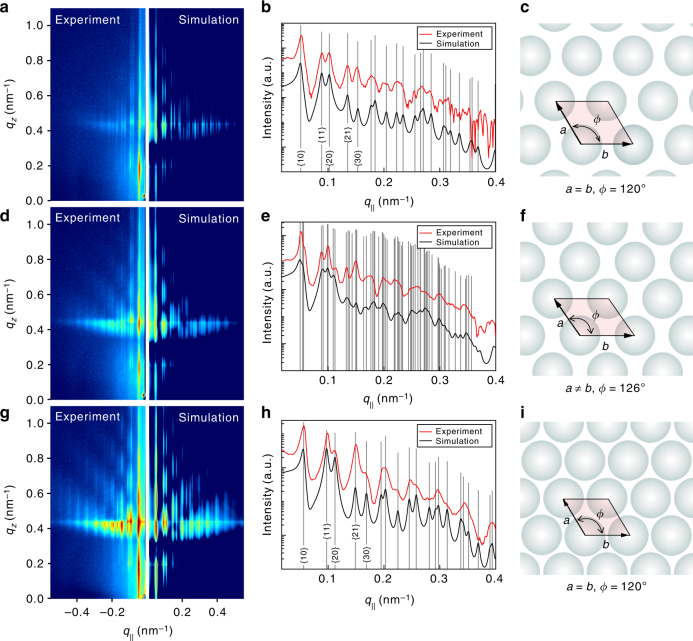
GISAXS data analyses and in-plane superlattice structures. **a**, **d**, **g** Experimental GISAXS patterns (left) compared with their corresponding simulated patterns (right). The GISAXS data are recorded at the surface pressures of 0.2, 10.4, and 17.6 mN m^−1^ and the corresponding areas per particle of 2.82 × 10^−2^, 2.30 × 10^−2^, and 2.13 × 10^−2^ μm^2^, respectively, (marked as diamonds in Fig. 1c). **b**, **e**, **h** The experimental (red solid lines) and simulated (black solid lines) 1D scattering profiles are extracted from the corresponding GISAXS patterns (i.e*.,* **a**, **d**, **g**), along the *q*_∥_ direction around *q*_*z*_ = 0.434 nm^−1^ (the Yoneda peak of water). Each gray line identifies a Bragg diffraction peak. **c**, **f**, **i** Schematic diagrams of the superlattice structures obtained from the theoretical simulations

As shown in Fig. 3a, the simulated 2D GISAXS pattern (right panel) agrees very well with the experimental data (left panel), where the intensity oscillations along the $q_z$ direction and the Bragg diffraction rods along the $q_\parallel$ direction are all well reproduced. The line cut in Fig. 3b is taken along the $q_\parallel$ direction around $q_z = 0.434\,{\mathrm{nm}}^{ - 1}$ (the Yoneda peak of water), where the scattering is most intensified. The ratios of the Bragg diffraction peak positons with respect to the fundamental one are $q_{\{ hk\} }/q_{\{ 10\} } \approx 1:\sqrt 3 :\sqrt 4 :\sqrt 7 :\sqrt 9$…, consistent with a 2D hexagonal structure. The Miller indices of the corresponding superlattice facets are {1,0}, {1,1}, {2,0}, {2,1}, {3,0} …, as designated in Fig. 3b. By fitting to the GISAXS data, the unit cell constants of the 2D superlattice are given as *a* = *b* = 142.3 nm and $\phi = 120^\circ$ (Fig. 3c). The interparticle distance is apparently larger than the PNS diameter, so it is a metastable state where the PNSs are likely trapped in a potential well located apart from the deep primary minimum due to van der Waals attraction at short range. In Fig. 3d, the simulated GISAXS pattern is compared with the experimental data, and the 1D line cut at *q*_*z*_ = 0.434 nm^−1^ is given in Fig. 3e. The Bragg peaks of different orders are marked with the gray lines at $q_\parallel = 0.050,0.055,0.056,0.088,0.091\,{\mathrm{nm}}^{ - 1}$ and so on. As revealed by the fitting results, the diffraction features identify the oblique superlattice structure with the unit cell constants $a = 138.9\,{\mathrm{nm}},b = 143.1\,{\mathrm{nm}}$, and $\phi = 126.4^\circ$ (Fig. 3f). With reference to Fig. 3a, this structure is resulted from the distortion of the metastable 2D hexagonal structure with increasing surface pressures. Nevertheless, the PNSs at this state are likely trapped in the same potential well. Figure 3g shows the experimental and simulated GISAXS patterns, where the main scattering features are all well reproduced. The 1D line cuts are plotted with the Bragg peaks marked (Fig. 3h). The associated superlattice structure is unveiled by the distinct pattern of the Bragg peaks. Evidently, it has the hexagonal close-packed structure with the superlattice constants *a* = *b* = 129.6 nm and$\phi = 120^\circ$ (Fig. 3i), where the interparticle distance is comparable to the PNS diameter. Due to external compression, the PNSs at the interface are able to overcome the repulsive energy barrier and fall irreversibly into the deep potential well created by van der Waals attraction and form the stable monolayer. The structural parameters obtained from the simulations are summarized in Supplementary Table 1.

### Out-of-plane structure analysis

So far, the in situ GISAXS technique is employed to probe the in-plane structure and track the dynamic self-assembly process of interfacial colloids in real time. Since the GISAXS signal is proportional to both $P\left( {q_\parallel ,q_z} \right)$ and ${\mathrm{S}}\left( {q_\parallel ,q_z} \right)$, it is also sensitive to the change in $P\left( {q_\parallel ,q_z} \right)$, which is related to the particle position at the interface. A quantitative analysis of the GISAXS patterns allows accurate determination of the immersion depth (or contact angle) of PNSs. For a single PNS particle adsorbed at the air/water interface, the X-ray scattering patterns are simulated for different immersion depths (*H*) (see Supplementary Fig. 6 and Supplementary Note 2). It is evident that small variation in *H* will cause pronounced changes to the scattering pattern. This is ascribed to the little electron density difference between PNSs and water. The part of PNSs immersed in water is almost “invisible” to the incident X-ray beam, therefore the part exposed in air constitutes the main scattering signal (~1000 times more than the part immersed in water).

**Fig. 4:**
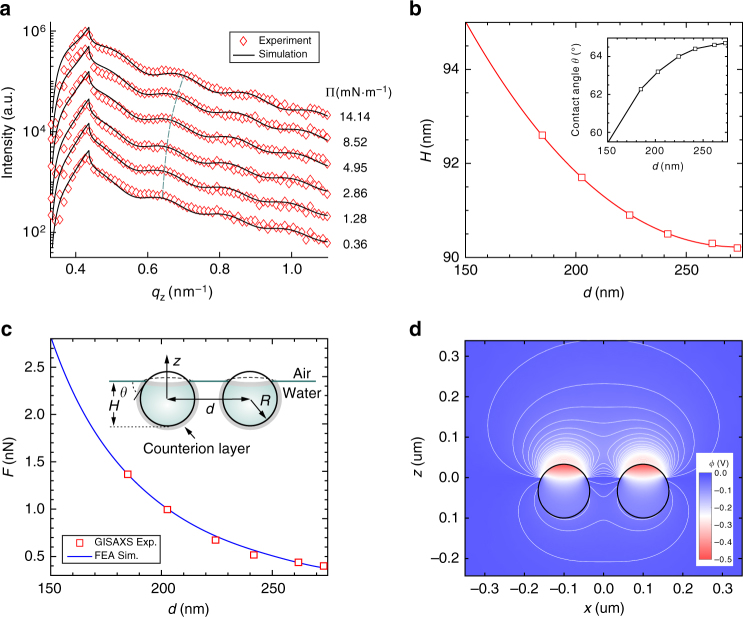
Out-of-plane structures and electrostatic interactions. **a** The 1D X-ray scattering profiles extracted along the *q*_*z*_ direction at the peak positions of the GISAXS patterns during stage II at surface pressures of 0.36, 1.28, 2.86, 4.95, 8.52, and 14.14  mN  m^−1^ (red diamonds). The theoretical simulations are given as black solid lines and the corresponding parameters are listed in Supplementary Table 2. **b** The immersion depth *H* and contact angle $\theta$ (inset) plotted as functions of the interparticle distance *d*. The lines are the polynomial fittings to the data as guides to the eye. **c** The magnitude of the out-of-plane component of the electrostatic fore plotted as a function of *d*. The electrostatic forces are estimated from surface tension analyses (red squares) and the numerical simulation results are obtained from FEA (blue line). Inset: schematic illustration of a pair of PNSs at the air/water interface with all the relevant parameters shown. **d** The electrostatic potential distribution of a pair of PNSs at the air/water interface with the equipotential lines displayed in white. The simulation is carried out using the same set of parameters as given in Supplementary Note 4. The colloidal particles (solid circles) have the radius *R* = 63.2 nm, the immersion depth *H* = 91.7 nm, and their separation *d* = 200 nm

During the in situ GISAXS experiments, we find a weak scattering peak appears and shifts away from the beamstop as the surface pressure increases at stage II (Supplementary Fig. 7). In the process of PNS assembling at the air/water interface, there are generally two kinds of states coexist before the final stable 2D hexagonal close-packed superlattice monolayer forms. The PNSs are unassembled or appear as small clusters (such as dimers and trimmers) in one state and form metastable islands or large ordered pieces in the other state. This weak scattering peak is originated from the PNSs in the first state. Figure 4a shows the 1D scattering profiles extracted at the corresponding peak positions along the $q_z$ direction at Π of 0.36, 1.28, 2.86, 4.95, 8.52, and 14.14 mN m^−1^, respectively. At this stage, the continuous compression from the LB barriers forces the PNSs to approach to each other, so the interparticle distance (*d*) decreases with increasing surface pressures. There are obvious shifts of the oscillation peaks and valleys of the 1D scattering curves toward the larger $q_z$ as Π increases, indicating the change of *H*. The PNS immersion depth *H* obtained by fitting the scattering curves is plotted as a function of *d* determined by the central positions of the scattering peaks (Fig. 4b). As *d* decreases from 272 to 184 nm, *H* increases from 90.2 to 92.7 nm. The corresponding contact angle ($\theta$) variation with *d* is shown in the inset, according to the relationship:${\mathrm{cos}}\left( \theta \right) = H/R - 1$.

For an isolated PNS adsorbed at the air/water interface, the surface energy per particle is estimated to be $E_{\mathrm{s}} \approx 10^5k_{\mathrm{B}}T$ (see Supplementary Fig. 8), where $k_{\mathrm{B}}T$ is the thermal energy and *k*_B_ is the Boltzmann constant^19^. The gravitational energy is given by $E_{\mathrm{g}} = \frac{4}{3}\pi R^4\rho g \approx 10^{ - 4}k_{\mathrm {B}}T$ for $\rho = 1.04$ g cm^−3^ and *R* = 63.2 nm. Therefore, both the thermal and gravitational energies are negligible when compared with the surface energy. The PNSs are essentially trapped in the surface energy well much deeper than $k_{\mathrm{B}}T$. By expressing the surface energy in terms of *H*, the force due to surface tension can be derived as $F_{\mathrm{s}} = 2{\mathrm{\pi }}\gamma (H - H_0)$ (see Supplementary Note 3), where *γ* is the surface tension of the air/water interface and *H*_0_ = 89.2 nm is the equilibrium immersion depth of an isolated particle. The *H*_0_ value is obtained asymptotically by extending the polynomial fitting curve to large *d* values in Fig. 4b. The direction of *F*_s_ is pointed upwards and perpendicular to the interface by symmetry consideration. If the PNS is forced into water deviating from its equilibrium position, *F*_s_ will neutralize the external force and restore balance. Consider a pair of PNSs at the air/water interface as depicted in the inset of Fig. 4c, when *d* decreases, *H* increases and so does *F*_s_; effectively, there is a force (denoted as *F*_e_) pushes the PNSs into water whose magnitude increases as particles get closer. Using the relation between *H* and *d* (Fig. 4b), $F_{\mathrm{s}}\left( { = F_{\mathrm{e}}} \right)$ can be plotted as a function of *d* (red squares in Fig. 4c). Reviewing all the forces exerting on PNSs, the electrostatic interaction is the only viable source for *F*_e_, while the possible thermal and gravitational origins of *F*_e_ are ruled out by its *d* dependence, in addition to their minimal scales.

To find the physical origin and evaluate the electrostatic interaction, we perform the finite-element analysis (FEA) by numerically solving the nonlinear Poisson-Boltzmann equation for a set of PNSs adsorbed at the air/water interface^17,25,27,38^ (see Supplementary Note 4 for details). We assume that the part of PNSs immersed in water carries a uniform layer of surface charge $\sigma _0$ and the part exposed in air retains a smaller amount of surface charge with its density proportional to $\sigma _0$^18,22,39^. The surface potential of the air/water surface is fixed at −50 mV^40^ and a uniform counterion (“Stern”) layer surrounds the part of PNSs immersed in water. The large mismatch in dielectric constants (${\mathrm{\varepsilon }}_{{\mathrm{air}}} \approx 1$ and ${\mathrm{\varepsilon }}_{{\mathrm{water}}} \approx 80$) crossing the interface results in asymmetric distributions of the electric fields as shown in Fig. 4d for a pair of PNSs. Applying the *H* and *d* values acquired from the GISAXS measurements, the electrostatic forces are computed by integrating over all the components of the electric forces in one direction acting on the surface charges of a particle in the fields created by other particles. The in-plane component of the electrostatic force accounts for the repulsive Coulomb interaction also predicted by the classical Derjaguin-Landau-Verwey-Overbeek (DLVO) theory, and the out-of-plane component directed from air to water is the origin of the force *F*_e_ that presses PNSs into water. As shown in Fig. 4c, a good agreement is achieved between the experimental and simulation results.

### Interaction potential

**Fig. 5:**
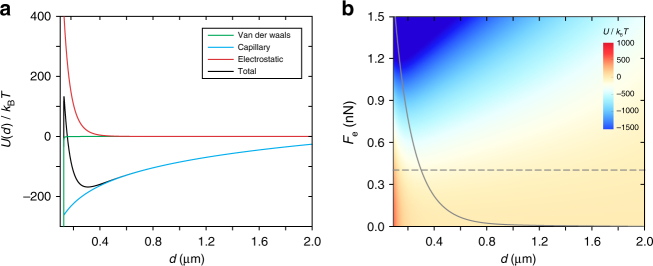
Estimation of the interaction potentials for a pair of PNSs. **a** The representative interaction potentials for a pair of PNSs adsorbed at the air/water interface at *F*_e_ = 0.4 nN. The total interaction potential per particle *U*(*d*), shown as a function of the interparticle distance *d* (black line), includes the van der Waals potential, the electrostatic potential and the capillary potential. **b** *U*(*d*) is plotted as a function of *F*_e_. The black solid line traces the positons of the secondary potential minima and the black dashed line marks for *F*_e_ = 0.4nN (as shown in **a**). The simulation details are given in the Supplementary Note 5

The electrostatic force *F*_e_ applied on PNSs is balanced by the surface tension, which creates a dimple in the water surface. The shape of the dimple is governed by the Young-Laplace equation^41^. By solving it in the cylindrical coordinates, the resulting water level around an isolated PNS can be written as $h\left( r \right) = \left( {F_{\mathrm{e}}/2\pi \gamma } \right)ln\left( {r/r_{\mathrm{c}}} \right)$, where *r*_c_ is the radius of the three-phase contact line. If the dimples around two PNSs overlap, they will induce capillary attraction and give rise to an interparticle interaction energy $U_{{\mathrm{cap}}}\left( d \right) = \left( {F_{\mathrm{e}}^2/2{\mathrm{\pi }}\gamma } \right)ln\left( {d/d_0} \right)$, where *d*_0_ is the gravitational capillary length^42,43^. Owing to its logarithmic nature, the capillary interaction can persist for a long range. Up to this point, a sketch of the full interparticle interaction potential per particle *U*(*d*) can be outlined by including the electrostatic, van der Waals and capillary interaction potentials (see Supplementary Note 5). As shown in Fig. 5a, *U*(*d*) is simulated for a pair of PNSs at the air/water interface for *F*_e_ = 0.4 nN. As can be seen, the capillary force is the origin of the attractive interaction between the like-charged interfacial colloids. The influence of *F*_e_ on *U*(*d*) and the shape of the secondary potential well is further investigated in Fig. 5b, where the trajectory of the potential minima is marked. Clearly, the interparticle distance at the secondary potential minimum decreases as *F*_e_ increases, which agrees well with the results obtained from the GISAXS measurements.

## Discussion

In conclusion, the unprecedented in situ X-ray scattering observation of the structure, kinetics, and evolution of interfacial colloids allows us to follow the key processes of the 2D crystallization and obtain critical information for interparticle interactions. Under continuous compression, the PNSs on the air/water interface of the LB trough initially form small clusters, then coalesce into metastable islands of 2D hexagonal superlattices, later become oblique structures under anisotropic interactions, and finally overcome the repulsive barrier and form the stable hexagonal close-packed superlattice monolayer. The unexpected discovery of the immersion depth variation with interparticle distances leads up to unveil the haunting mystery of the attractive interaction between like-charged interfacial particles. Detailed interaction analyses and numerical simulations demonstrate the electrostatic force from neighboring particles presses the colloids into water, which deforms the interface and induces the long-range capillary attraction. Given the many useful, interesting and economically important implementations of colloidal suspensions, the current work has significance beyond its immediate application to the 2D interfacial self-assembly.

## Methods

### Materials

PNSs in deionized water (resistivity $\sim 18.2\,{\mathrm{M\Omega }} \,{\mathrm{cm}}^{{\mathrm{ - 1}}}$) were purchased from Hugebio with the weight percentage of 5 wt%. The PNSs have anionic sulfate groups on their surfaces to make them negatively charged in water. The PNS solution was diluted with the mixed solution of ethanol and water (volume ratio 6:4:10) and ultrasonicated for 10 min before used. All the water used in this work was deionized.

### Zeta potential and surface charge density

The electrophoretic mobility and zeta potential of the PNSs were measured with a 90Plus particle size analyzer in combination with Zeta PALS software from Brookhaven Instruments. The mobility of the PNSs in deionized water was −2.76 µm cm Vs^−1^, corresponding to the zeta potential of −41.1 mV using the algorithm of O’Brien and White. The surface charge density of the PNSs is negative and estimated to be *σ*_0_ = 1.34 μC cm^−2^.

### Langmuir-Blodgett isotherm surface pressure

The surface pressure-particle area isotherm plot was recorded on a KSV NIMA 2002 trough (7.5 × 36.4 cm^2^, *W* × *L*) equipped with two movable barriers and one dip coater. The surface pressure was measured using a tensiometer (i.e., a platinum Wilhelmy plate) held by a microelectronic system. The subphase used in the experiments was deionized water. Before the experiment, the trough was cleaned twice with alcohol and once with deionized water. Residual impurities were cleaned from the air/water interface by surface suction. The good baseline in the isotherms confirmed the interface cleanliness. Then, the PNS solution (~0.2 mL) was carefully dropped onto the water surface through a tilted silicon slide. After the surface stabilized, the PNSs were compressed through the barriers at a speed of 0.3 mm min^−1^ and the isotherm was recorded and the interface area was reduced from 250 to 30 cm^2^. In addition, the surface pressure measurements of the supernatant solution obtained via centrifugation of the PNS solution were carried out (equal volume of the supernatant solution was used as that in the GISAXS experiments but without PNSs). The resultant surface pressure curve was compared with the baseline (Supplementary Fig. 10), which verified there was no detectable impurity in the solution that would affect the experimental results.

### Small-angle X-ray scattering measurements

SAXS measurements were conducted at the beamline BL16B of Shanghai Synchrotron Radiation Facility (SSRF), with the incident X-ray photon energy of 10 keV (i.e., the wavelength *λ* = 1.239 Å). The flux of the incident X-ray beam was about 2 × 10^11^ photons s^−1^. The PNS solution was sealed in a square cell (1.5 mm in thickness) with two Kapton windows. The sample-to-detector distance was set to 5120.0 mm. The signal was collected through a Rayonix SX-165 CCD (Rayonix, Evanston, IL, USA) area detector with 2048 × 2048 pixels and each pixel size of 80 × 80 μm^2^. The scattering intensities were integrated into 1D scattering curves *I*(*q*) as a function of the modulus of the scattering vector q, where $q = (4{\mathrm{\pi }}/\lambda ){\mathrm{sin}}(\theta /2)$, and *λ* and $\theta$ were the incident X-ray wavelength and the scattering angle, respectively.

### Grazing-incidence small-angle X-ray scattering experiments

GISAXS measurements were performed at the beamline BL16B of SSRF. The incident X-ray beam had a downward inclination of 0.367° after passing through the focusing mirror. The wavelength of the incident X-ray was 1.239 Å, corresponding to 10 KeV in photon energy. The flux of the incident X-ray was about 2 × 10^11^ photons s^−1^. The LB device was placed on a vibration isolation platform as shown in Supplementary Fig. 1. The distance between the LB device and the CCD detector was set to 5120.0 mm. After the LB was set-up, the time-resolved GISAXS data were collected during the dynamic compression process. Notably, the geometric footprint of the beam (0.4 × 0.5 mm^2^,*V* × *H*) along the incident direction is 5.67 cm and the total illuminated area is estimated to be 2.84 cm^2^. The detected X-ray scattering signal is the sum of the scattering intensities from all the PNSs in the illuminated area.

### Ex situ SEM imaging

The silicon wafers of size 20 × 20 mm^2^ were used as substrates for SEM characterizations. The substrates were sonicated for 10 min in water and then in ethanol and dried under the N_2_ flow. Before the LB barriers compressed, the wafers were firstly immersed in water. When the isothermal surface pressures reached the same values as those in the corresponding GISAXS measurements, the wafers were lifted off and dried naturally. The scanning electron microscopy (SEM, JEOL 7800 Prime or FEI nanoSEM 450) was used to character the transferred PNSs. The acceleration voltage of the electron was set to 2.0 kV.

### SAXS and GISAXS data analyses

SAXS and GISAXS data analyses were completed based on the self-developed codes in Matlab^@^. The experimental scattering patterns were also calibrated and formatted with these codes. The data were fitted through the covariance matrix adaptation evolution strategy (CMA-ES) method^44^. All the GISAXS patterns including the experiments and simulations were presented on a base-10 logarithmic scale.

### Data availability

The authors declare that all data supporting the findings of this study are available within the manuscript and the Supplementary Information or from the corresponding author upon request.

## Electronic supplementary material

Supplementary Information

Description of Additional Supplementary Files

Supplementary Movie 1
