# Supplementary material for: In situ X-ray scattering observation of two-dimensional interfacial colloidal crystallization
Source: Nat Commun. 2018 Apr 6;9:1335. doi: 10.1038/s41467-018-03767-y (PMC5889402; doi:10.1038/s41467-018-03767-y)
Supplement: Supplementary file 1 — Supplementary Information [file 41467_2018_3767_MOESM1_ESM.pdf]

Supplementary Information for

In situ X-ray scattering observation of two-dimensional interfacial colloidal crystallization

Longlong Wu, Xiao Wang, Geng Wang, and Gang Chen*

*Corresponding author. Email: gchen@shanghaitech.edu.cn (G.C.)

Supplementary Figures

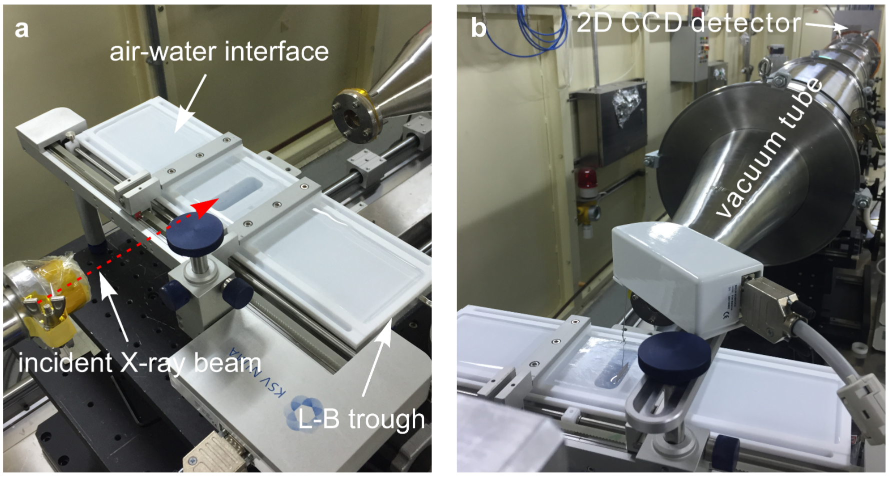

Supplementary Figure 1 | Photographs of the GISAXS experiment setup. a, side view and **b,** top view. The X-ray beam enters from the right. The 2D CCD detector is placed at the end of the vacuum tube.

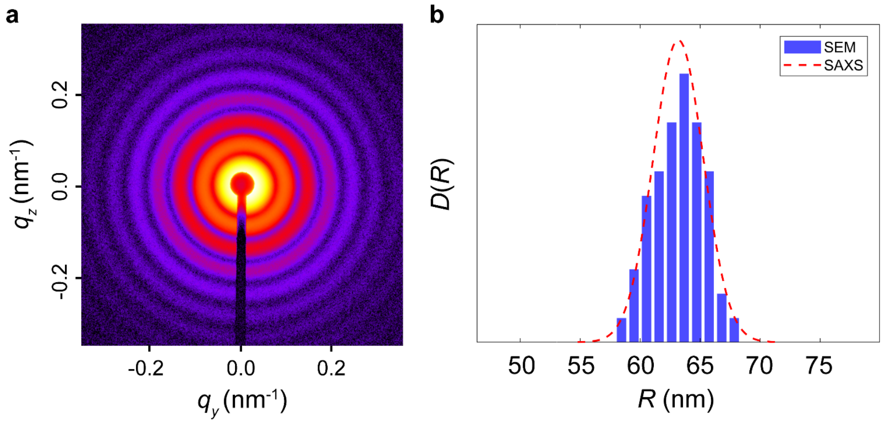

Supplementary Figure 2 | SAXS and SEM Characterizations of the PNSs. a, Two-dimensional SAXS pattern of the PNSs in solution. **b,** Comparison of the PNS size distributions obtained from SEM measurements and SAXS data fittings.

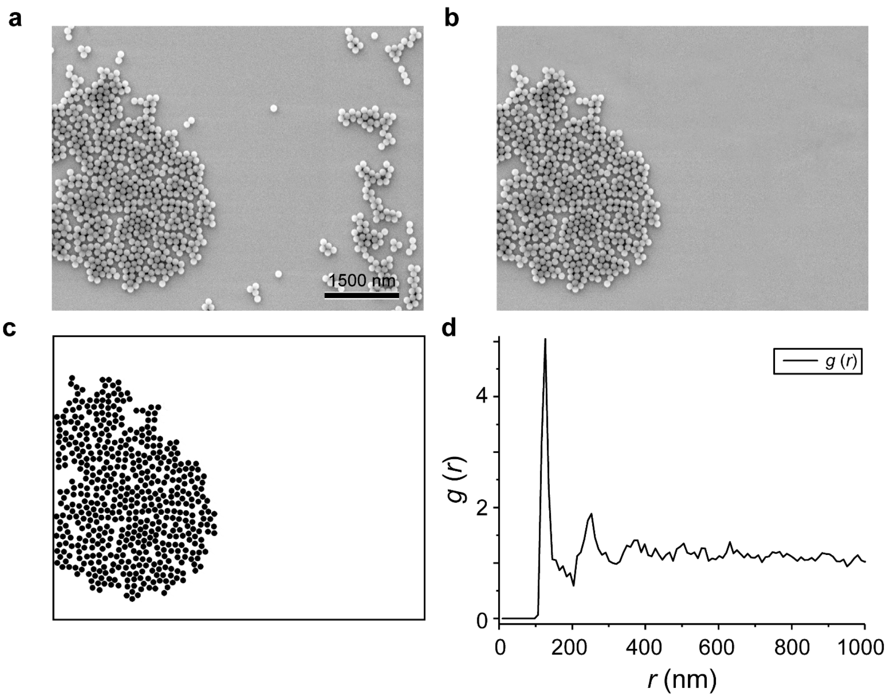

Supplementary Figure 3 | Radial distribution function $g(r)$ for the PNSs trapped at the interface extracted from the ex situ SEM measurement. a, The original SEM image. **b,** The isolated island from (a). **c,** Binarization of the image (b). **d**, Extracted radial distribution function $g(r)$ from the image (c).

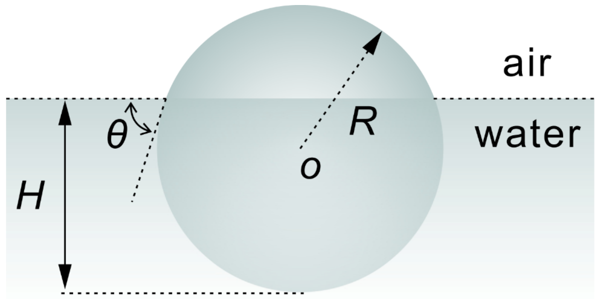

Supplementary Figure 4 | Schematic illustration of a single colloidal sphere trapped at the air/water interface. H is the immersion depth. θ is the contact angle, defined as $\cos\theta = H/R - 1$. R is the radius of PNSs.

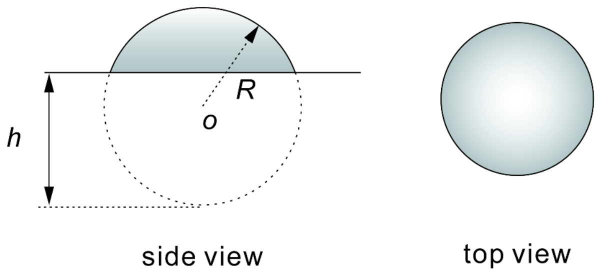

Supplementary Figure 5 | Sketch of a truncated sphere with the side and top views, respectively. *R* is the radius of the full sphere. *h* is the height of the truncated part.

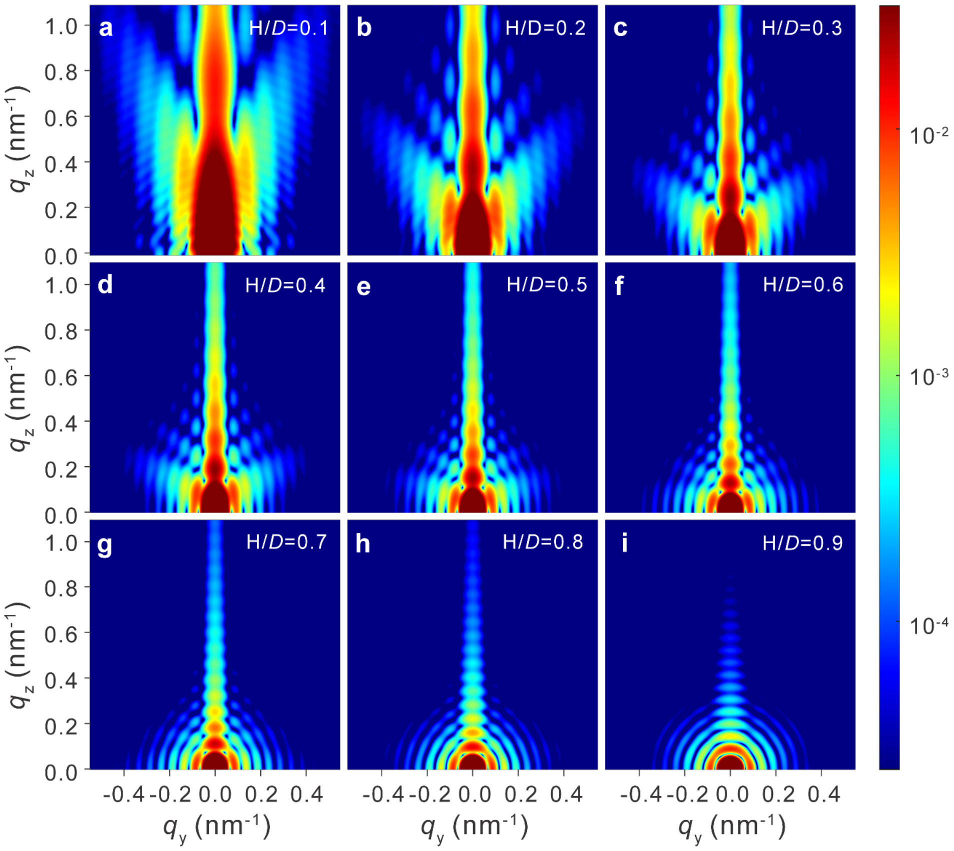

Supplementary Figure 6 | 2D SAXS patterns of a single colloidal particle at the air/water interface by varying its immersion depth *H*. As shown in the figures, *H* is normalized to the diameter *D* of PNSs (*H*/*D*) for **a,** 0.1, **b,** 0.2, **c,** 0.3, **d,** 0.4, **e,** 0.5, **f,** 0.6, **g,** 0.7, **h,** 0.8 and **i,** 0.9. *D* equals to 126.4 nm.

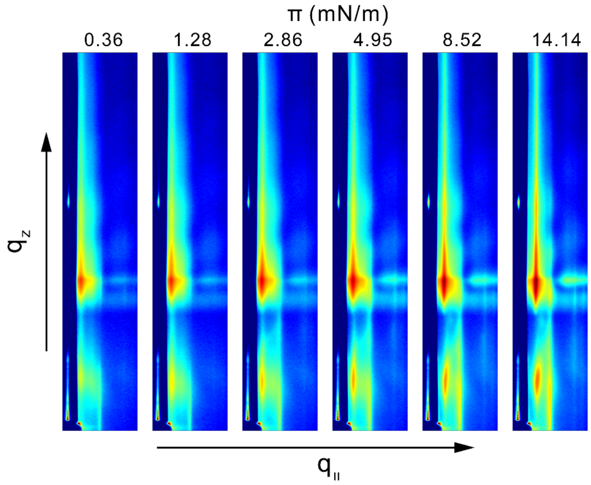

Supplementary Figure 7 | In situ GISAXS pattern evolution as the surface pressure increases. The corresponding surface pressures are given at the top of each GISAXS scattering pattern. These data were recorded in the same time as those shown in Figure 2 of the main text and during the same in situ GISAXS experiment.

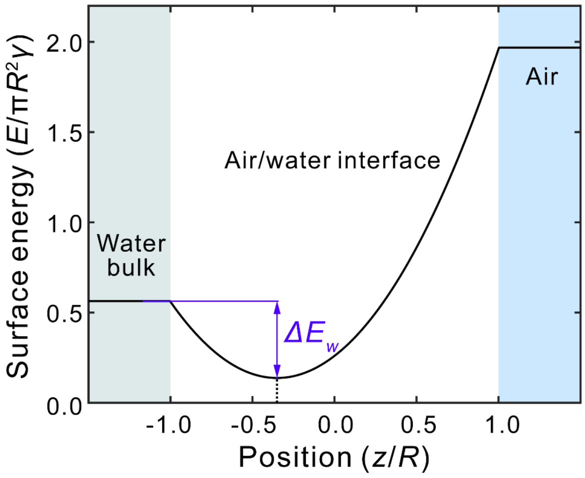

Supplementary Figure 8 | Estimation of surface energy well for an isolated colloid. The energy analysis is based on the model proposed by Pieranski. γ is the surface tension of water and z is the central positon of the colloid. Note that *z/R* = -1 corresponds to a sphere immersed in water, and that the *z/R* = +1 corresponds to a sphere in air. For a colloid of 63.2 nm in radius, its immersion depth is estimated to be 85.32 nm. The energy barrier ΔE_{w}, as marked in the figure, is about 4.6×10^{-16} J, which is much greater than the thermal fluctuation $k_{\mathrm{B}}T \approx 4.12 \times 10^{-21}$ J. Thus, the adsorption of colloids at the air/water interface is permanent and irreversible. In addition, the dish line indicates the minimum energy for an isolated colloid at the air/water interface.

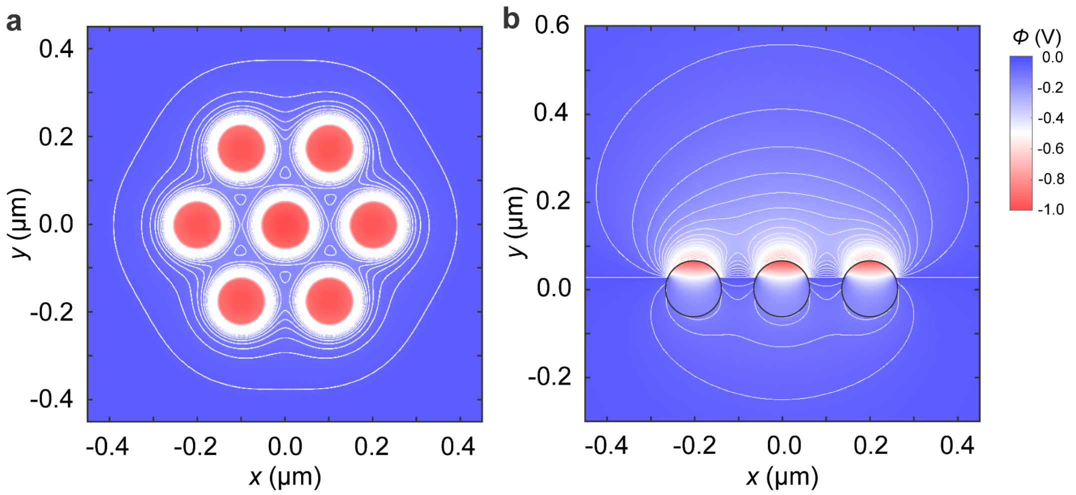

Supplementary Figure 9 | The electrostatic potential distributions of a PNS with six nearest neighbors. **a,** Horizontal cross-sectional view of the electrostatic potential distribution extracted at z = 14 nm above the air/water interface. **b,** Vertical cross-sectional view of the electrostatic potential distribution extracted at y = 0 nm. The distance between the PNSs is 200 nm. The white lines represent the equipotential lines.

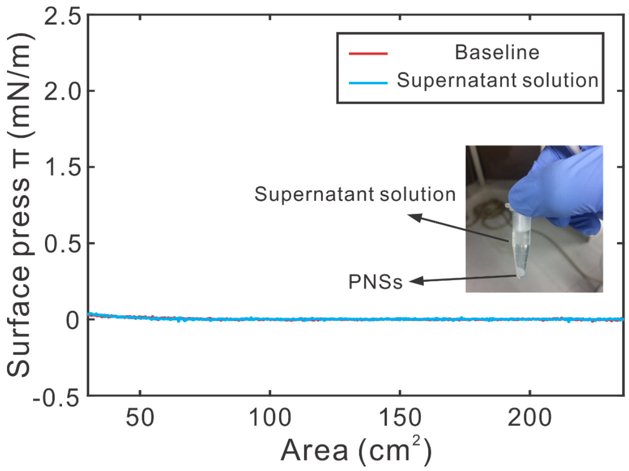

Supplementary Figure 10 | Comparison of the surface pressure between the deionized water (baseline) and the supernatant solution obtained from the PNS solution but without PNSs. The supernatant solution was obtained via centrifugation of the PNS solution as shown by the photo in the inset.

Supplementary Table

Supplementary Table 1 | Structural parameters obtained by fitting the 2D GISAXS patterns of the PNSs at the air/water interface (the incident angle $\alpha_i = 0.367°$). σ is the r.m.s roughness of the air/water interface. a, b and ϕ are the unit cell parameters. $\bar{R}$ represents the mean value of the colloidal radius. H is the immersion depth. δ_D is the relative r.m.s displacement. N_n is the average number of colloids in the coherent region. z is the width parameter for the PNS size distribution. δ is the Lorentzian parameter.

	σ (Å)	a (nm)	b (nm)	ϕ (°)	$\bar{R}$(nm)	H(nm)	δ_D	N_n	z	δ
Fig.3a	3.1	142.3	142.3	120.0	63.2	95.0	0.03	21083	1334	0.03
Fig.3d	4.0	138.9	143.1	126.4	63.2	95.4	0.03	22007	1334	0.07
Fig.3g	4.6	129.6	129.6	120.0	63.2	97.0	0.03	22090	1334	0.058

Supplementary Table 2 | Structural parameters obtained from fitting the 1D scattering profiles extracted from the GISAXS patterns along the q_z direction. σ is the r.m.s roughness of the interface. $\bar{R}$ represents the mean value of the colloidal radius. z is the width parameter for the colloidal size distribution. H is the corresponding immersion depth. Π represents the surface pressure.

q_y (nm^{-1})	σ (nm)	$\bar{R}$(nm)	z	H(nm)	Π ($mN \cdot m^{-1}$)
0.023	0.80	63.2	1334	90.2	0.36
0.024	0.60	63.2	1334	90.3	1.28
0.026	1.10	63.2	1334	90.6	2.86
0.028	1.00	63.2	1334	91.1	4.95
0.031	1.30	63.2	1334	91.7	8.52
0.034	1.28	63.2	1334	92.6	14.14
0.056	1.31	63.2	1334	97.0	17.60

Supplementary Notes

Supplementary Note 1 | Modeling of GISAXS profiles for the two-dimensional system at an interface. Under the distorted-wave Born approximation (DWBA)[1], the scattering intensity for a two-dimensional system at grazing-incidence geometry is the summation of all the intensities over the x-y plane:

$$I_{\mathrm{GISAXS}}(\mathbf{q}_{\parallel}, k_{iz}, k_{fz}) = \frac{k_c C}{4\pi r^2} \left|T_i(k_{iz})\right|^2 \left|T_f(k_{fz})\right|^2 \times \sum_{n=1}^{N} \sum_{n'=1}^{N} \mathrm{F}_{n'}^{*}(\mathbf{q}_{\parallel}, k_{iz}, k_{fz}) \mathrm{F}_n(\mathbf{q}_{\parallel}, k_{iz}, k_{fz}) \times \mathrm{e}^{-j\mathbf{q}_{\parallel} \cdot (\mathbf{R}_n - \mathbf{R}_{n'})}, \quad (1)$$

where $\mathrm{F}_n(\mathbf{q}_{\parallel}, k_{iz}, k_{fz})$ and $\mathbf{R}_n$ are the scattering amplitude and position of the nth particle, respectively. $\mathbf{q}_{\parallel}$ is the in-plane component of the wavevector transfer, $|\mathbf{q}_{\parallel}| = q_{\parallel} = \sqrt{q_x^2 + q_y^2}$ and q_z is the out-of-plane component with $q_z = k_{fz} - k_{iz}$. k_{iz} and k_{fz} are the z components of the wave vectors of the incoming and outgoing X-ray beams, respectively. k_c is the vertical component of the momentum vector at the critical angle of total external reflection, with $k_c^2 = k_0^2(1 - n^2)$, where $k_0 = 2\pi/\lambda$ and n is the refraction index for the incident X-ray. C is the illuminated area. r is the distance between the scattering center and the detector. T_i and T_f are respectively the modified Fresnel transmission coefficients of the incident and reflected waves:

$$T_{i,f} = \frac{2k_z}{k_z + \tilde{k}_z} \mathrm{e}^{-\frac{\sigma^2 \left(\tilde{k}_z - k_z\right)^2}{2}} . \tag{2}$$

Here, k_z and $\tilde{k}_z$ are the vertical components of the wave vectors in the air and "substrate", respectively, given as $k_z = -\sqrt{k_0^2 - \left|k_{\|}\right|^2}$ and $\tilde{k}_z = \sqrt{n^2 k_0^2 - \left|\tilde{k}_{\|}\right|^2}$. σ is the r.m.s roughness of the interface. For a crystal-like lattice of 2D monolayer particles with the domains rotating freely in the x-y plane and their relative orientations being uncorrelated, the scattering from these domains can be written as:

$$\begin{aligned} I_{\mathrm{GISAXS}}(q_{\|}, k_{iz}, k_{fz}) &= \frac{k_c C}{4\pi r^2} \left|T_i\left(k_{iz}\right)\right|^2 \left|T_f\left(k_{fz}\right)\right|^2 \times \left\langle \left| \sum_{n=1}^{N_n} \exp^{j\mathbf{q}_{\|}\cdot\mathbf{r}_n} \sum_{m=1}^{N_m} \mathrm{F}_p(\mathbf{q}_{\|}, k_{iz}, k_{fz}) \times \mathrm{e}^{j\mathbf{q}_{\|}\cdot\mathbf{r}_m} \right|^2 \right\rangle_{\mathrm{o}} \\ &= \frac{k_c C}{4\pi r^2} \left|T_i\left(k_{iz}\right)\right|^2 \left|T_f\left(k_{fz}\right)\right|^2 \times \left\langle \left| \sum_{n=1}^{N_n} A_u\left(\mathbf{q}_{\|}, k_{iz}, k_{fz}\right) \times \mathrm{e}^{j\mathbf{q}_{\|}\cdot\mathbf{r}_n} \right|^2 \right\rangle_{\mathrm{o}} \end{aligned} , \tag{3}$$

where $A_{\mathrm{u}}\left(\mathbf{q}_{\|}, k_{iz}, k_{fz}\right)$ is the effective form factor of the unit cell of these domains which is given as:

$$A_{\mathrm{u}}\left(\mathbf{q}_{\|}, k_{iz}, k_{fz}\right) = \sum_{m=1}^{N_m} F_{\mathrm{p}}(\mathbf{q}_{\|}, k_{iz}, k_{fz}) \times \mathrm{e}^{j\mathbf{q}_{\|}\cdot\mathbf{r}_m} . \tag{4}$$

Here, N_n is the number of the identical unit cells in the sample and N_m is the number of the particles in the unit cell. $\mathbf{r}_n$ is the central position of the unit cell and $\mathbf{r}_m$ is the position of the particle in the cell. The angle brackets $\langle\ \rangle_{\mathrm{o}}$ denotes the orientational average. In addition, the polydispersity of the particles will change the scattering intensity. Assuming that the properties of a particle are not correlated to its position[2-4], so that equation (S3) can be simplified as:

$$\begin{aligned} I_{\mathrm{GISAXS}}(q_{\|}, k_{iz}, k_{fz}) &= \frac{k_c C}{4\pi r^2} \left|T_i\left(k_{iz}\right)\right|^2 \left|T_f\left(k_{fz}\right)\right|^2 \times \left\langle \sum_{n=1}^{N_n} \sum_{n'=1}^{N_n} \left\langle A_{\mathrm{u}}\left(\mathbf{q}_{\|}, k_{iz}, k_{fz}\right) A_{\mathrm{u}'}^{*}\left(\mathbf{q}_{\|}, k_{iz}, k_{fz}\right) \right\rangle_{\mathrm{d}} \times \mathrm{e}^{j\mathbf{q}_{\|}\cdot(\mathbf{r}_n - \mathbf{r}_{n'})} \right\rangle_{\mathrm{o}} \\ &= \frac{k_c C}{4\pi r^2} \left|T_i\left(k_{iz}\right)\right|^2 \left|T_f\left(k_{fz}\right)\right|^2 \times \\ &\left\langle \sum_{n=1}^{N_n} \sum_{n'=1}^{N_n} \left[\left| \left\langle A_{\mathrm{u}}\left(\mathbf{q}_{\|}, k_{iz}, k_{fz}\right) \right\rangle_{\mathrm{d}} \right|^2 + \left[\left\langle \left| A_{\mathrm{u}}\left(\mathbf{q}_{\|}, k_{iz}, k_{fz}\right) \right|^2 \right\rangle_{\mathrm{d}} - \left| \left\langle A_{\mathrm{u}}\left(\mathbf{q}_{\|}, k_{iz}, k_{fz}\right) \right\rangle_{d} \right|^2 \right] \delta_{nn'} \right] \times \mathrm{e}^{j\mathbf{q}_{\|}\cdot(\mathbf{r}_n - \mathbf{r}_{n'})} \right\rangle_{\mathrm{o}} \\ &= \frac{k_c C}{4\pi r^2} \left|T_i\left(k_{iz}\right)\right|^2 \left|T_f\left(k_{fz}\right)\right|^2 \times \\ &\left\langle \sum_{n=1}^{N_n} \sum_{n'=1}^{N_n} \left[\left| \left\langle A_{\mathrm{u}}\left(\mathbf{q}_{\|}, k_{iz}, k_{fz}\right) \right\rangle_{\mathrm{d}} \right|^2 \times \mathrm{e}^{j\mathbf{q}_{\|}\cdot(\mathbf{r}_n - \mathbf{r}_{n'})} + \left\langle \left| A_{\mathrm{u}}\left(\mathbf{q}_{\|}, k_{iz}, k_{fz}\right) \right|^2 \right\rangle_{\mathrm{d}} - \left| \left\langle A_{\mathrm{u}}\left(\mathbf{q}_{\|}, k_{iz}, k_{fz}\right) \right\rangle_{d} \right|^2 \right] \right\rangle_{\mathrm{o}} \\ &= \frac{k_c C}{4\pi r^2} \left|T_i\left(k_{iz}\right)\right|^2 \left|T_f\left(k_{fz}\right)\right|^2 \times \left\langle \left| A_{\mathrm{u}}\left(\mathbf{q}_{\|}, k_{iz}, k_{fz}\right) \right|^2 \right\rangle_{\mathrm{od}} \\ &\left[\frac{1}{\left\langle \left| A_{\mathrm{u}}\left(\mathbf{q}_{\|}, k_{iz}, k_{fz}\right) \right|^2 \right\rangle_{\mathrm{od}}} \left\langle \sum_{n=1}^{N_n} \sum_{n'=1}^{N_n} \left| \left\langle A_{\mathrm{u}}\left(\mathbf{q}_{\|}, k_{iz}, k_{fz}\right) \right\rangle_{d} \right|^2 \times \mathrm{e}^{j\mathbf{q}_{\|}\cdot(\mathbf{r}_n - \mathbf{r}_{n'})} \right\rangle_{\mathrm{o}} + 1 - \frac{\left| \left\langle A_{\mathrm{u}}\left(\mathbf{q}_{\|}, k_{iz}, k_{fz}\right) \right\rangle_{\mathrm{od}} \right|^2}{\left\langle \left| A_{\mathrm{u}}\left(\mathbf{q}_{\|}, k_{iz}, k_{fz}\right) \right|^2 \right\rangle_{\mathrm{od}}} \right] \\ &= \frac{k_c C}{4\pi r^2} \left|T_i\left(k_{iz}\right)\right|^2 \left|T_f\left(k_{fz}\right)\right|^2 P\left(q_{\|}, k_{iz}, k_{fz}\right) \left[\frac{z_0(q_{\|}, k_{iz}, k_{fz})}{P\left(q_{\|}, k_{iz}, k_{fz}\right)} + 1 - \beta\left(q_{\|}, k_{iz}, k_{fz}\right) \right]. \end{aligned} \tag{5}$$

Here, the angle brackets $\langle\ \rangle_{\mathrm{d}}$ represents the intensity average over the particle polydispersity. $\delta_{nn'}$ is the Kronecker delta function. $P(q_{\|},k_{iz},k_{fz})$ is defined as the orientationally averaged form factor including the polydispersity of the particle: $P(q_{\|},k_{iz},k_{fz}) = \left\langle \left| A_u\left(\mathbf{q}_{\|},k_{iz},k_{fz}\right)\right|^2 \right\rangle_{\mathrm{od}}$. $\beta(q_{\|},k_{iz},k_{fz})$ is expressed as:

$$\beta\left(q_{\|},k_{iz},k_{fz}\right) = \frac{\left|\left\langle F(q_{\|},k_{iz},k_{fz})\right\rangle_{\mathrm{od}}\right|^2}{\left\langle \left|F(q_{\|},k_{iz},k_{fz})\right|^2\right\rangle_{\mathrm{od}}}. \tag{6}$$

With references to previous studies[3-5], $Z_0\left(q_{\|},k_{iz},k_{fz}\right)$ is defined as the lattice factor (assuming no disorder), which can be simplified as:

$$\begin{aligned} Z_0\left(q_{\|},k_{iz},k_{fz}\right) &= \left\langle \sum_{n=1}^{N_n}\sum_{n'=1}^{N_n} \left|\left\langle A_u\left(\mathbf{q}_{\|},k_{iz},k_{fz}\right)\right\rangle_d\right|^2 \times \mathrm{e}^{j\mathbf{q}_{\|}\cdot(\mathbf{r}_n-\mathbf{r}_{n'})} \right\rangle_{\mathrm{o}} \\ &= \frac{N_n}{2\pi q_{\|,hk} v}\sum_{\{hk\}}^{m_{hk}}\left|\sum_{p=1}^{N_p} F_p\left(\mathbf{q}_{\|,hk},k_{iz},k_{fz}\right)\mathrm{e}^{2\pi j\left(x_p h+y_p k\right)}\right|^2 \times L(q_{\|}-q_{\|,hk}) \end{aligned}, \tag{7}$$

where v is the area of the 2D unit cell, m_{hk} is the multiplicity of the reflection hk , corresponding to the reciprocal space at $\mathbf{q}_{\|,hk}$. L is the peak-shape function. Here, the Lorentzian peak shape is used, given as $L(q_{\|}-q_{\|,hk}) = \delta/2\pi \Big/ \left[\left(q_{\|}-q_{\|,hk}\right)^2 - \left(\delta/2\right)^2\right]$ and δ is the full width at half maximum (FWHM) of the peak at $q_{\|,hk}$. Since the fluctuation (*e.g.* thermal fluctuation) of the particle positions may result in lattice disorder, the Debye-Waller factor is introduced:[4]

$$G(q) = \exp\left(-\sigma_D^2 \Lambda^2 q_{\|}^2\right), \tag{8}$$

where $\sigma_D = \sigma_{\mathrm{rms}}/\Lambda$ is the relative r.m.s displacement for a lattice of size Λ . Finally, the scattering intensity is expressed as:

$$\begin{aligned} I_{\mathrm{GISAXS}}(q_{\|},k_{iz},k_{fz}) &= \frac{k_{\mathrm{c}}C}{4\pi r^2}\left|T_i\left(k_{iz}\right)\right|^2\left|T_f\left(k_{fz}\right)\right|^2 P(q_{\|},k_{iz},k_{fz})\left[\frac{z_0(\mathbf{q}_{\|},k_{iz},k_{fz})}{P\left(\mathbf{q}_{\|},k_{iz},k_{fz}\right)}G(q_{\|}) + 1 - \beta\left(q_{\|},k_{iz},k_{fz}\right)G(q_{\|})\right]. \\ &= \frac{k_{\mathrm{c}}C}{4\pi r^2}\left|T_i\left(k_{iz}\right)\right|^2\left|T_f\left(k_{fz}\right)\right|^2 P(q_{\|},k_{iz},k_{fz})S(q_{\|},k_{iz},k_{fz}). \end{aligned} \tag{9}$$

Now, $S(q_{\|},k_{iz},k_{fz})$ is effectively a revised definition of the structure factor, which includes disorder from polydispersity and positional fluctuations:

$$S(q_{\|},k_{iz},k_{fz}) = \frac{Z_0(\mathbf{q}_{\|},k_{iz},k_{fz})}{P\left(\mathbf{q}_{\|},k_{iz},k_{fz}\right)}G(q_{\|}) + 1 - \beta\left(q_{\|},k_{iz},k_{fz}\right)G(q_{\|}). \tag{10}$$

Supplementary Note 2 | Form factor for the PNSs adsorbed at the air/water interface. As illustrated in Supplementary Fig. 4, for a colloidal sphere adsorbed at the interface, its X-ray scattering amplitude can be reduced as:

$$
\begin{aligned}
F(q_{\|},k_{iz},k_{fz},R,H) &= \frac{\rho_{\mathrm{c}}\mathrm{V}_{\mathrm{a}}F_{\mathrm{a}}\left(\mathrm{q}_{\|},k_{iz},k_{fz},R,H\right)+(\rho_{\mathrm{c}}-\rho_{\mathrm{w}})\mathrm{V}_{\mathrm{b}}F_{\mathrm{b}}\left(\mathrm{q}_{\|},k_{iz},k_{fz},R,H\right)}{\rho_{c}\mathrm{V}_{a}+(\rho_{c}-\rho_{w})\mathrm{V}_{b}} \\
&= \frac{(\rho_{\mathrm{c}}-\rho_{\mathrm{w}})V_{\mathrm{s}}F_{\mathrm{s}}\left(\mathrm{q}_{\|},k_{iz},k_{fz},R\right)+\rho_{\mathrm{w}}\left[V_{\mathrm{s}}F_{\mathrm{s}}\left(\mathrm{q}_{\|},k_{iz},k_{fz},R,H\right)-V_{\mathrm{b}}F_{\mathrm{b}}\left(\mathrm{q}_{\|},k_{iz},k_{fz},R,H\right)\right]}{(\rho_{\mathrm{c}}-\rho_{\mathrm{w}})V_{\mathrm{s}}+\rho_{\mathrm{w}}\left(V_{\mathrm{s}}-V_{\mathrm{b}}\right)}, \\
&= \frac{\Delta\rho V_{\mathrm{s}}F_{\mathrm{s}}\left(\mathrm{q}_{\|},k_{iz},k_{fz},R\right)+\rho_{\mathrm{w}}V_{\mathrm{a}}F_{\mathrm{a}}\left(\mathrm{q}_{\|},k_{iz},k_{fz},R,H\right)}{\Delta\rho V_{\mathrm{s}}+\rho_{\mathrm{w}}V_{\mathrm{a}}}
\end{aligned}
\tag{11}
$$

where ρ_{c} and ρ_{w} are the electron densities of the PNSs and water, respectively ($\rho_{\mathrm{c}}=0.34\, e^{-}\cdot\text{Å}^{-3},\rho_{\mathrm{w}}=0.33\, e^{-}\cdot\text{Å}^{-3}$) and $\Delta\rho=\rho_{\mathrm{c}}-\rho_{\mathrm{w}}$. V_{s} is the volume of PNSs, V_{a} is the portion of the volume exposed in air and V_{b} is the volume immersed in water. $F_{\mathrm{s}}\left(q_{\|},q_{z},R\right)$, $F_{\mathrm{a}}\left(q_{\|},q_{z},R,H\right)$ and $F_{\mathrm{b}}\left(q_{\|},q_{z},R,H\right)$ are the corresponding form factors described by the full and truncated spheres, respectively. $F_{\mathrm{s}}\left(q_{\|},q_{z},R\right)$ is expressed as:

$$
F_{\mathrm{s}}(q_{\|},q_{z},R)=\frac{\sin\left(qR\right)-qR\cos\left(qR\right)}{(qR)^{3}}. \tag{12}
$$

As illustrated in Supplementary Fig. 5, the form factor $F_{a}\left(q_{\|},q_{z},R,h\right)$ for a truncated sphere is given as:

$$
\begin{aligned}
F_{\mathrm{a}}\left(\mathbf{q},R,h\right) &= \frac{1}{V_{sp}}\int_{h-R}^{R}2\pi R_{z}^{2}\frac{J_{1}\left(q_{\|}R_{z}\right)}{q_{\|}R_{z}}e^{iq_{z}z}dz \\
R_{z} &= \sqrt{R^{2}-z^{2}} \qquad , \\
V_{\mathrm{sp}} &= \pi R^{3}\left[\frac{2}{3}+\frac{R-h}{R}-\frac{1}{3}\left(\frac{R-h}{R}\right)^{3}\right]
\end{aligned}
\tag{13}
$$

where V_{sp} is the volume of the truncated sphere.

Therefore, the $P\left(q_{\|},k_{iz},k_{fz}\right)$ function is given as:

$$
\begin{aligned}
P\left(q_{\|},k_{iz},k_{fz}\right) &= \frac{1}{2\pi}\iint D\left(R\right)\left|V(R)F\left(q_{\|}\cos\left(\phi\right),q_{\|}\sin\left(\phi\right),k_{iz},k_{fz},R,H\right)\right|^{2}d\phi dR \\
&= \int D\left(R\right)\left|V(R)F\left(q_{\|},k_{iz},k_{fz},R,H\right)\right|^{2}dR
\end{aligned}
. \tag{14}
$$

Here, $D(R)$ is the Schultz distribution with two parameters:

$$
D(R)=\left[\frac{z+1}{\bar{R}}\right]^{z+1}\frac{R^{z}}{\Gamma(z+1)}\exp\left[-\left(z+1\right)\frac{R}{\bar{R}}\right], \tag{15}
$$

where $\bar{\mathrm{R}}$ is the mean value of the particle radius R and z is a width parameter. $\Gamma\left(\mathrm{x}\right)$ is the Gamma function. Thus, the $\beta\left(q_{\|},k_{iz},k_{fz}\right)$ is given as:

$$
\beta\left(q_{\|},k_{iz},k_{fz}\right)=\frac{\left|\int D\left(R\right)V(R)F\left(q_{\|},k_{iz},k_{fz},R,H\right)dR\right|^{2}}{\int D\left(R\right)\left|V(R)F\left(q_{\|},k_{iz},k_{fz},R,H\right)\right|^{2}dR}. \tag{16}
$$

Supplementary Note 3 | Surface tension force for PNSs. As shown in Supplementary Fig. 4, the trapping energy for a colloidal particle at the air/water interface can be expressed as[6]:

$$\begin{aligned} E_{\mathrm{p}} &= 2\pi R^2\gamma(1-\cos\theta)\cos\theta_0 + 2\pi R\tau\sin\theta - \pi R^2\gamma\cos^2\theta \\ &= 2\pi R^2\gamma\left[1-\left(\frac{H}{R}-1\right)\right]\left(\frac{H_0}{R}-1\right) + 2\tau\pi R\sqrt{1-\left(\frac{H}{R}-1\right)^2} - \pi R^2\gamma\left(\frac{H}{R}-1\right)^2, \end{aligned} \tag{17}$$

where H is the immersion depth of the particle, H_0 is the equilibrium immersion depth, γ is the surface tension for the air/water interface, τ is the line tension, associated with the excess free energy at the three-phase contact line. Taking the derivative with respect to H, we get:

$$\begin{aligned} \frac{dE_{\mathrm{p}}}{dH} &= -2\pi\gamma R\left(\frac{H_0}{R}-1\right) - 2\pi\tau\left(\frac{H}{R}-1\right)\frac{1}{\sqrt{1-\left(\frac{H}{R}-1\right)^2}} - 2\pi\gamma R\left(\frac{H}{R}-1\right) \\ &= -2\pi\gamma(H+H_0-2R) + 2\pi\tau\frac{\frac{H}{R}-1}{\sqrt{1-\left(\frac{H}{R}-1\right)^2}} \end{aligned}. \tag{18}$$

The line tension contribution is small enough to be neglected in our experiment, so the force to move a colloidal particle from its position H_0 to H is given as:

$$\begin{aligned} F &= -\left[\frac{dE_{\mathrm{p}}}{dH}(H) - \frac{dE_{\mathrm{p}}}{dH}(H_0)\right]. \\ &= 2\pi\gamma(H-H_0) \end{aligned} \tag{19}$$

Supplementary Note 4 | Finite-element analysis (FEA). The FEA was performed through COMSOL@ (Version 5.3), to investigate the electrostatic interactions between PNSs based on a 3D model, by solving the classical nonlinear Poisson-Boltzmann equation[7-10]:

$$\nabla^2\phi = -\sum_{i=1}^{n}\frac{z_i e}{\varepsilon}N_i\,\mathrm{e}^{-\frac{z_i e\phi}{k_{\mathrm{B}}T}}. \tag{20}$$

Here, N_i is the ion number density in unit volume, z_i is the corresponding charge number of the ion, e is the electronic charge, k_{B} is the Boltzmann constant, and T is the temperature which equals to 298 K in this case. In the bulk water solution, the ion densities for H^+ and OH^- are close to $10^{-7}\mathrm{mol}\cdot\mathrm{L}^{-1}$. The corresponding dielectric constant for water ε_{w} is estimated to 80. According to the recent experiment and theoretical study[9,11], the air/water interface maintains a constant negative potential at -50 mV. A uniform counterion layer surrounds the part of PNSs immersed in water and its thickness is taken as 1 nm.[10] In addition, colloidal particles (126.4 nm in diameter) with their dielectric constant $\varepsilon_{\mathrm{p}} = 2$, separated by the distances obtained from the GISAXS simulation results, were placed at

the air/water interface. The diffuse layer around the particles was about 80 nm in thickness. The boundaries of the simulation box were grounded. For the parts of particles immersed in the water, they carried a uniform surface charge density $\sigma_{\text{water}} = -1.34\ \mu\text{C}\cdot\text{cm}^{-2}$. However, the parts exposed in air retained a tiny amount of surface charge density σ_{air}[8,12]. The system was divided into more than 5,000,000 triangular elements. The interactions between the PNSs in the vertical direction (*i.e.* along the *z* direction) were obtained by integrating over the vertical components of the electrostatic forces (F_z). By taking into account the electric fields created by six nearest neighbors and tuning the ratio between σ_{air} and σ_{water}, the best fit to the data in Fig. 4c was obtained for the ratio of 0.0021. As one would expect, the fraction of surface charge density at the air-particle side will be further reduced if we consider the next-nearest neighbors and the next-next-nearest neighbors, … etc. Here, the six nearest neighbors are considered to keep the computation task within a tolerable scale while retain a clear physical picture with viable parameters. The corresponding electrostatic potential distributions are shown in Supplementary Fig. 9.

Supplementary Note 5 | Interaction potential for PNSs adsorbed at the air/water interface. For the pairwise interaction potential $U(d)$ of PNSs per particle at the air/water interface, it includes the attractive van der Waals potential $U_{\text{vdW}}(d)$, the capillary potential $U_{\text{cap}}(d)$, and the repulsive electrostatic potential $U_{\text{el}}(d)$:

$$U(d) = U_{\text{vdW}}(d) + U_{\text{cap}}(d) + U_{\text{el}}(d). \tag{21}$$

Here, the attractive van der Waals potential is expressed as:[13]

$$U_{\text{vdW}}(d) = -\frac{A_{\text{eff}}}{6(d-R_1-R_2)}\frac{R_1R_2}{(R_1+R_2)}, \tag{22}$$

where A_{eff} is an effective Hamaker constant[14,15]. Due to the PNS strides the air/water interface, the effective Hamaker constant is given as:

$$A_{\text{eff}} = A_{\text{v}} + f^2(3-2f)(A_{\text{w}} - A_{\text{v}}), \tag{23}$$

where A_{v} and A_{w} are the Hamaker constant for PNSs in vacuum and water, seperately. f is the corresponding volume fraction for PNS immersed in water, which is given as:

$$\begin{aligned} f &= \frac{V_{\text{s}} - V_{\text{sp}}}{V_{\text{s}}} \\ &= \frac{\frac{4}{3}\pi R^3 - \pi R^3\left[\frac{2}{3} + \frac{R-h}{R} - \frac{1}{3}\left(\frac{R-h}{R}\right)^3\right]}{\frac{4}{3}\pi R^3} \\ &= \frac{1}{2} - \frac{3}{4}\left(1-\frac{H}{R}\right) + \frac{1}{4}\left(1-\frac{H}{R}\right)^3. \end{aligned} \tag{24}$$

In equation (S24), V_s is the volume of the PNS and V_{sp} is the part of the PNS exposed in air, as given in equation (S13). H is the immersion depth of the PNS and equals to h. Thus, the Hamaker constant is estimated to $A_{eff} = 1.53 \times 10^{-20}$ J.

The capillary attraction potential is given as:[16,17]

$$U_{cap}(d) = \frac{F_e^2}{2\pi\gamma} ln(d/d_0), \tag{25}$$

where F_e is the force pulling the particle into water, γ is the surface tension of the water, which equals to $\gamma = 72$ mN/m, and d_0 is the gravitational capillary length[17], which is taken as 2.7 mm.

For the charged particles at an interface, they interact through both the water and the air, yielding an electrostatic potential per particle:[13,15,18]

$$U_{el}(d) = \frac{Z_1 Z_2}{4\pi d \varepsilon_0 \varepsilon}\left(\frac{\varepsilon^2}{\varepsilon^2 - 1}\exp(-\kappa d) + \frac{1}{\varepsilon \kappa^2 d^2}\right), \tag{26}$$

where ε_0 is the permittivity of the air, which equals to $\varepsilon_0 = 8.85 \times 10^{-12}\ \mathrm{F \cdot m^{-1}}$ and κ is the inverse of the Debye length[18], taken as $12.5\ \mu\mathrm{m}^{-1}$. d is the interparticle distance, ε is the relative dielectric constant of water and Z is the effective charge number. In Eq. S25, the first term is the screened Coulomb repulsion between two charged particles and the second term represents the dipolar repulsive interaction. However, the most part of the PNS is immersed in water and the part exposed in air carries a tiny surface charge. Therefore, Z, is estimated as:[12]

$$Z = 2\pi R \sin\theta \kappa^{-1} \sigma_{water} \alpha_{water}, \tag{27}$$

where R is the radius of the PNS and θ is the corresponding contact angle, which equals to $\theta = \arccos(\frac{R}{H} - 1)$. In addition, α_{water} is the degree of dissociation of the sulfate groups at the particle-water surface, which is estimated to be 0.25.[6] Finally, the effective charge number per particle is estimated as $Z_1 = Z_2 = 752.9\ e$.

Supplementary References

1 Renaud, G., Lazzari, R. & Leroy, F. Probing surface and interface morphology with Grazing Incidence Small Angle X-Ray Scattering. *Surf. Sci. Rep.* **64**, 255-380, doi:10.1016/j.surfrep.2009.07.002 (2009).

2 Kotlarchyk, M. & Chen, S. H. Analysis of small angle neutron scattering spectra from polydisperse interacting colloids. *The Journal of chemical physics* **79**, 2461-2469 (1983).

3 Forster, S. *et al.* Scattering curves of ordered mesoscopic materials. *J. Phys. Chem. B* **109**, 1347-1360, doi:10.1021/jp0467494 (2005).

4 Senesi, A. J. & Lee, B. Small-angle scattering of particle assemblies. *J. Appl. Crystallogr.* **48**, 1172-1182, doi:10.1107/S1600576715011474 (2015).

5 Yager, K. G., Zhang, Y., Lu, F. & Gang, O. Periodic lattices of arbitrary nano-objects: modeling and applications for self-assembled systems. *J. Appl. Crystallogr.* **47**, 118-129, doi:10.1107/s160057671302832x (2013).
6 Maestro, A., Guzman, E., Ortega, F. & Rubio, R. G. Contact angle of micro- and nanoparticles at fluid interfaces. *Current Opinion in Colloid & Interface Science* **19**, 355-367, doi:10.1016/j.cocis.2014.04.008 (2014).
7 Barnes, G. & Gentle, I. *Interfacial science: an introduction*. (Oxford University Press, 2011).
8 Bossa, G. V., Roth, J., Bohinc, K. & May, S. The apparent charge of nanoparticles trapped at a water interface. *Soft Matter* **12**, 4229-4240, doi:10.1039/c6sm00334f (2016).
9 Shrestha, A., Bohinc, K. & May, S. Immersion depth of positively versus negatively charged nanoparticles at the air-water interface: a Poisson-Boltzmann model. *Langmuir* **28**, 14301-14307, doi:10.1021/la303177f (2012).
10 Masschaele, K., Park, B. J., Furst, E. M., Fransaer, J. & Vermant, J. Finite ion-size effects dominate the interaction between charged colloidal particles at an oil-water interface. *Phys. Rev. Lett.* **105**, 048303, doi:10.1103/PhysRevLett.105.048303 (2010).
11 Gehring, T. & Fischer, T. M. Diffusion of Nanoparticles at an Air/Water Interface Is Not Invariant under a Reversal of the Particle Charge. *The Journal of Physical Chemistry C* **115**, 23677-23681, doi:10.1021/jp2061738 (2011).
12 Aveyard, R. *et al.* Measurement of long-range repulsive forces between charged particles at an oil-water interface. *Phys. Rev. Lett.* **88**, 246102, doi:10.1103/PhysRevLett.88.246102 (2002).
13 Ghezzi, F. & Earnshaw, J. C. Formation of meso-structures in colloidal monolayers. *J Phys-Condens Mat* **9**, L517-L523, doi:Doi 10.1088/0953-8984/9/37/004 (1997).
14 Williams, D. F. & Berg, J. C. The Aggregation of Colloidal Particles at the Air-Water-Interface. *J. Colloid Interface Sci.* **152**, 218-229, doi:Doi 10.1016/0021-9797(92)90021-D (1992).
15 Bresme, F. & Oettel, M. Nanoparticles at fluid interfaces. *J Phys Condens Matter* **19**, 413101, doi:10.1088/0953-8984/19/41/413101 (2007).
16 Smalyukh, II *et al.* Ordered droplet structures at the liquid crystal surface and elastic-capillary colloidal interactions. *Phys. Rev. Lett.* **93**, 117801, doi:10.1103/PhysRevLett.93.117801 (2004).
17 Oettel, M. & Dietrich, S. Colloidal interactions at fluid interfaces. *Langmuir* **24**, 1425-1441, doi:10.1021/la702794d (2008).
18 Hurd, A. J. The electrostatic interaction between interfacial colloidal particles. *J. Phys. A: Math. Gen.* **18**, L1055-L1060, doi:10.1088/0305-4470/18/16/011 (1985).
