# Supplementary material for: In situ X-ray scattering observation of two-dimensional interfacial colloidal crystallization
Source: Nat Commun. 2018 Apr 6;9:1335. doi: 10.1038/s41467-018-03767-y (PMC5889402; doi:10.1038/s41467-018-03767-y)
Supplement: Supplementary file 2 — Description of Additional Supplementary Files [file 41467_2018_3767_MOESM2_ESM.pdf]

Description of Additional Supplementary Files

File Name: Supplementary Movie 1
Description: The in situ X-ray scattering patterns recorded during the self-assembly process of colloids at the air/water interface of a Langmuir-Blodgett trough.
